# Characteristics and Lenvatinib Treatment Response of Unresectable Hepatocellular Carcinoma with Iso-High Intensity in the Hepatobiliary Phase of EOB-MRI

**DOI:** 10.3390/cancers13143633

**Published:** 2021-07-20

**Authors:** Akinori Kubo, Goki Suda, Megumi Kimura, Osamu Maehara, Yoshimasa Tokuchi, Takashi Kitagataya, Masatsugu Ohara, Ren Yamada, Taku Shigesawa, Kazuharu Suzuki, Naoki Kawagishi, Masato Nakai, Takuya Sho, Mitsuteru Natsuizaka, Kenichi Morikawa, Koji Ogawa, Shunsuke Ohnishi, Naoya Sakamoto

**Affiliations:** 1 Departments of Gastroenterology and Hepatology, Graduate School of Medicine, Hokkaido University, Sapporo 060-8589, Japan; kubo.akinori.q5@elms.hokudai.ac.jp (A.K.); myamakam@med.hokudai.ac.jp (M.K.); tokuchi.yoshimasa.e2@elms.hokudai.ac.jp (Y.T.); t.kitagataya@pop.med.hokudai.ac.jp (T.K.); masamasama_zu@yahoo.co.jp (M.O.); renyama5@pop.med.hokudai.ac.jp (R.Y.); Tshigesawa@pop.med.hokudai.ac.jp (T.S.); kazuharu-s@hospital.hakodate.hokkaido.jp (K.S.); naopaleg@yahoo.co.jp (N.K.); mnakai@pop.med.hokudai.ac.jp (M.N.); shotaku@pop.med.hokudai.ac.jp (T.S.); mitsuteru@natsuizakaclinic.com (M.N.); kenichi.morikawa@med.hokudai.ac.jp (K.M.); k-ogawa@med.hokudai.ac.jp (K.O.); sakamoto@med.hokudai.ac.jp (N.S.); 2 Laboratory of Molecular and Cellular Medicine, Faculty of Pharmaceutical Sciences, Hokkaido University, Sapporo 060-8589, Japan; maeosa17@frontier.hokudai.ac.jp (O.M.); sonishi@pop.med.hokudai.ac.jp (S.O.)

**Keywords:** *CTNNB-1*, HCC with iso-high intensity in the hepatobiliary phase of EOB-MRI, Lenvatinib

## Abstract

**Simple Summary:**

HCC with alterations in *CTNNB1* (which encodes β-catenin) is resistant to immune checkpoint inhibitors and is associated with HCC with iso-high intensity in the hepatobiliary phase of EOB-MRI in resectable HCC. However, the prevalence, characteristics, mutation profile, and treatment response in *unresectable* HCC with iso-high intensity in the hepatobiliary phase of EOB-MRI are not well clarified. In this study, we showed that the prevalence was 13%, and the response to lenvatinib does not differ between HCC with and without iso-high intensity in the hepatobiliary phase of EOB-MRI. We analyzed *CTNNB-1* mutations using cell-free DNA, providing support for their association with iso-high intensity in the hepatobiliary phase of EOB-MRI.

**Abstract:**

In hepatocellular carcinoma (HCC), *CTNNB-1* mutations, which cause resistance to immune checkpoint inhibitors, are associated with HCC with iso-high intensity in the hepatobiliary phase of gadoxetic acid-enhanced magnetic resonance imaging (EOB-MRI) in resectable HCC; however, analyses on unresectable HCC are lacking. This study analyzed the prevalence, characteristics, response to lenvatinib, and *CTNNB-1* mutation frequency in unresectable HCC with iso-high intensity in the hepatobiliary phase of EOB-MRI. In 52 patients with unresectable HCC treated with lenvatinib, the prevalence of iso-high intensity in the hepatobiliary phase of EOB-MRI was 13%. All patients had multiple HCCs, and 3 patients had multiple HCCs with iso-high intensity in the hepatobiliary phase of EOB-MRI. Lenvatinib response to progression-free survival and overall survival were similar between patients with or without iso-high intensity in the hepatobiliary phase of EOB-MRI. Seven patients (three and four patients who had unresectable HCC with or without iso-high intensity in the hepatobiliary phase of EOB-MRI, respectively) underwent genetic analyses. Among these, two (67%, 2/3) who had HCC with iso-high intensity in the hepatobiliary phase of EOB-MRI carried a *CTNNB-1* mutation, while all four patients who had HCC without iso-high intensity in the hepatobiliary phase of EOB-MRI did not carry the *CTNNB-1* mutation. This study’s findings have clinical implications for the detection and treatment of HCC with iso-high intensity in the hepatobiliary phase of EOB-MRI.

## 1. Introduction

Recent advances have dramatically changed the hepatocellular carcinoma (HCC) treatment landscape. The development of immune checkpoint inhibitors (ICIs), which target negative anti-cancer immune response regulators, has drastically improved anti-cancer therapy. Various clinical trials and real-world data have revealed the efficacy of ICIs, conferring a durable response, which is not observed in conventional anti-cancer therapy, in various malignancies [1,2,3,4,5]. A clinical trial on HCC has revealed that bevacizumab plus atezolizumab has been associated with better overall survival (OS) in patients with unresectable tumors than sorafenib, which was the standard therapy for patients with unresectable HCC [6].

However, some patients did not experience sufficient treatment response. Thus, predictive factors for response to ICIs have been an important clinical issue and identified in various malignancies, including PD-L1 expression, immune cell infiltration, mutation burden, T cell receptor repertoires, and the gut microbiome [7]. Recently, Harding et al. reported that in patients with advanced HCC carrying WNT/β-catenin mutations, ICI treatment results in a lower disease control rate and shorter median OS than those for HCC without WNT/β-catenin mutations [8]. Thus, the presence of WNT/β-catenin mutations in advanced HCC might be an effective predictive factor for resistance to ICI therapy. However, analyses of genetic alterations in advanced HCC are difficult because biopsy of advanced HCC sometimes causes tumor dissemination and bleeding. Thus, alternative methods for the detection of WNT/β-catenin mutations in advanced HCC are required. HCC imaging using gadoxetic acid-enhanced magnetic resonance imaging (EOB-MRI) could be a candidate approach. In the hepatobiliary phase of EOB-MRI, HCC typically shows low intensity, while HCC with the WNT/β-catenin mutation shows iso-high intensity because WNT/β-catenin mutations result in higher organic anion transporter 1B3 (OATP1B3) expression than in surrounding non-HCC hepatocytes [9,10,11]. In resectable HCC, the reported frequency of cases with iso-high intensity in the hepatobiliary phase of EOB-MRI is 12% (22/180) to 20% (5/25) [12,13,14,15]. However, the prevalence of unresectable HCC with iso-high intensity in the hepatobiliary phase of EOB-MRI, requiring systemic therapy, has not been clarified. 

Analyses of cell-free DNA (cfDNA) are promising for detecting genetic alterations in HCC [16,17] and may provide insight into the frequent mutations in unresectable HCC with high intensity in the hepatobiliary phase of EOB-MRI.

Lenvatinib is a recently developed multi-kinase inhibitor that targets VEGFR1–3, FGFR1–4, RET c-Kit, and PDGFR-α mediated signaling [18,19]. The REFLECT trial, which is a phase 3 clinical trial regarding lenvatinib for patients with unresectable HCC, revealed that lenvatinib has similar OS compared with sorafenib, while lenvatinib has superior progression-free survival than sorafenib [20]. Real-world data validated the efficacy and safety of lenvatinib for patients with unresectable HCC [21,22]. Thus, in patients with unresectable HCC, who might have a poor response to ICIs, lenvatinib could be alternative first-line therapy. However, the efficacy and safety of lenvatinib in patients with unresectable HCC with iso-high intensity in the hepatobiliary phase of EOB-MRI have not been clarified.

Therefore, in this study, we analyzed (a) the prevalence of unresectable HCC with iso-high intensity in the hepatobiliary phase of EOB-MRI in patients with unresectable HCC who were treated with lenvatinib, (b) the response to lenvatinib, and (c) the relationship between the presence of unresectable HCC with iso-high intensity in the hepatobiliary phase of EOB-MRI and β-Catenin (*CTNNB-1*) mutations determined by a cfDNA analysis.

## 2. Materials and Methods

### 2.1. Patients and Study Design

Patients with unresectable HCC for whom lenvatinib treatment was initiated at Hokkaido University Hospital between April 2018 and April 2021 were screened in this retrospective study. We included patients if they had an EOB-MRI examination before lenvatinib treatment and were evaluated after treatment using dynamic computed tomography (CT) or EOB-MRI at baseline and every 2–3 months. Patients were excluded if they had decompensated liver cirrhosis, had insufficient clinical data or no EOB-MRI examination, had a treatment duration of less than 1 week, or were not evaluated for treatment responses by dynamic CT or MRI.

Attending physicians routinely assessed patients using laboratory tests and physical examinations every 2 weeks and using enhanced CT or EOB-MRI every 2–3 months after treatment initiation.

### 2.2. Treatment Protocol

Patients were administrated lenvatinib according to the package insert as follows: once-daily doses of 8 and 12 mg for patients weighing <60 and ≥60 kg, respectively.

### 2.3. Evaluation of HCC with Iso-High Intensity in the Hepatobiliary Phase of EOB-MRI

Two MRI systems, Achieva 1.5 T dStream (Philips N. V., Tokyo, Japan) and TRILLIUM–OVAL 3.0 T (HITACHI, Tokyo, Japan), were utilized for the evaluation of HCC according to previously described methods [10] by two hepatologists. The signal intensities (SIs) of the tumor and surrounding non-tumor liver tissues were measured by defining regions of interest (ROIs). The enhancement in HCC relative to liver parenchyma was calculated according to a previously described method [15] as follows. Relative intensity ratio (RIR) = SI (nodule)/SI (parenchyma). Relative enhancement ratio (RER) = RIR (in hepatobiliary-phase images)/RIR (precontract RIR). Tumor enhancement was defined as iso-high when RER ≥ 1.0.

### 2.4. Evaluation of the Response to Lenvatinib

Treatment responses were evaluated every 2–3 months using dynamic CT based on mRECIST criteria [23].

### 2.5. CTNNB1 Mutation Detection in Cell-Free DNA

Eleven of the 52 patients included had cfDNA at baseline and/or within 1 month after treatment initiation. Two of these patients had HCC with iso-high intensity in the hepatobiliary phase of EOB-MRI. Moreover, four patients without HCC with iso-high intensity in the hepatobiliary phase of EOB-MRI who initiated lenvatinib in June 2019 and agreed to cfDNA collection were selected for an analysis of *CTNNB1* alterations. In addition, *CTNNB1* alterations in cfDNA were analyzed in another patient who had unresectable HCC with iso-high intensity in the hepatobiliary phase of EOB-MRI and had cfDNA.

For the extraction of cfDNA, 4 mL of plasma was collected, and cfDNA was extracted using the AVENIO cfDNA Isolation Kit (Roche Diagnostics K. K., Tokyo, Japan) according to the manufacturer’s protocol at Tsukuba i-Laboratory LLP (Tsukuba, Ibaraki, Japan) and stored at −80 °C. The concentration of cfDNA was determined using the Qubit dsDNA HS Assay Kit (Thermo Fisher Scientific, Waltham, MA, USA), and fragment sizes were examined using the Agilent 2200 TapeStation System with High Sensitivity D5000 ScreenTape Assay (Agilent, Santa Clara, CA, USA).

We conducted library preparation and sequencing according to the manufacturer’s (Thermo Fisher Scientific) methods. We utilized an Oncomine Colon cfDNA for PCR amplification of target regions, and library preparation for sequencing reads was mapped using Torrent Suite 5.6.0 (Thermo Fisher Scientific). Variant calling was performed using Oncomine Colon Liquid Biopsy-w2.1-DNA—Single Sample workflow in Ion Reporter version 5.6 (Thermo Fisher Scientific). Modified Oncomine_Colon_cfDNA.03062017, a BED file, was used for variant calling at hotspot sites. Annotations of called variants were based on dbSNP151, CCDS (NCBI, Release 15), RefSeq (UCSC Genome Browser, Nov 2018), Gencode (UCSC Genome Browser, ver. 19), and 1000 Genomes (phase3 release v5).

### 2.6. Statistical Analysis

Categorical variables were analyzed using the chi-square and Fisher’s exact tests. Continuous variables were analyzed using the Mann–Whitney U-test. In this study, we set statistical significance at *p* < 0.05.

Progression-free survival (PFS) and overall survival (OS) were defined as follows; the time from the initiation of lenvatinib to the day of disease progression or last follow-up and death, respectively. PFS and OS curves were compared using the log-rank test. The association of etiology and overall survival was analyzed by Cox regression analysis. Statistical analyses were performed using IBM SPSS Statistics 22.0 (IBM Corp., Armonk, NY, USA).

## 3. Results

### 3.1. Enrolled Patients and Baseline Characteristics

**Figure 1:**
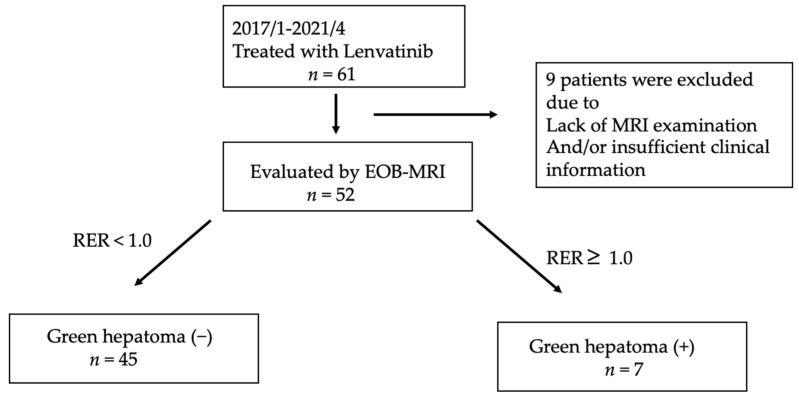
Study Flow.

Between April 2018 and April 2021, a total of 61 patients with unresectable HCC were treated with lenvatinib at Hokkaido University Hospital. Among these patients, nine were excluded owing to a lack of EOB-MRI examination and/or insufficient treatment duration. Finally, 52 patients with unresectable HCC were included in the study (Figure 1).

Table 1 shows the baseline characteristics of patients. The median age of patients with HCC with iso-high intensity in the hepatobiliary phase of EOB-MRI was 70 years and that of patients without was 71 years. A total of 88% of patients (46/52) were males. Forty-one and 11 patients had Child–Pugh grades A and B, respectively. Twenty-seven and 25 patients had BCLC stages B and C, respectively. The median serum AFP and PIVKA-II levels were 56.7 IU/mL (range, 1.6–449,909 IU/mL) and 404.0 mAU/mL (range, 13–416,670 mAU/mL), respectively. Regarding disease etiology, 14, 8, and 30 patients had HBV infection, HCV infection, and non-B non-C liver disease.

### 3.2. Prevalence and Characteristics of HCC with Iso-High Intensity in the Hepatobiliary Phase of EOB-MRI in Patients with Unresectable HCC Who Were Treated with Lenvatinib

**Figure 2:**
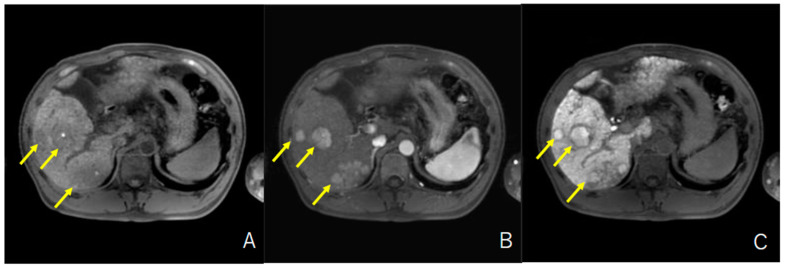
Typical MR images of unresectable HCC with iso-high intensity in the hepatobiliary phase. (**A**) Plain phase, (**B**) Arterial phase, (**C**) Hepatobiliary phase.

Overall, among 52 patients, 7 (13%) had HCC with high intensity in the hepatobiliary phase of EOB-MRI. Typical MR images are shown in Figure 2. As summarized in Table 1, the median age, sex, BCLC stage, and tumor markers were similar in patients with unresectable HCC with or without iso-high intensity in the hepatobiliary phase of EOB-MRI. Table 2 presents the characteristics of patients with unresectable HCC with iso-high intensity in the hepatobiliary phase of EOB-MRI. All patients had multiple HCCs and had both HCCs with or without iso-high intensity in the hepatobiliary phase of EOB-MRI simultaneously. Three patients (42.9%) had multiple HCCs with iso-high intensity in the hepatobiliary phase of EOB-MRI.

### 3.3. Treatment Response, Overall Survival, and Progression-Free Survival

Subsequently, we analyzed the response to lenvatinib, PFS, and OS in patients with unresectable HCC. As shown in Table 3, treatment responses were similar between patients with or without HCC with iso-high intensity in the hepatobiliary phase of EOB-MRI; the objective response rates (ORR) were 42.8% (3/7) and 48.8% (22/45), and the disease control rates (DCR) were 100% (7/7) and 86.6% (39/45) in patients with or without HCC with iso-high intensity in the hepatobiliary phase of EOB-MRI, respectively.

**Figure 3:**
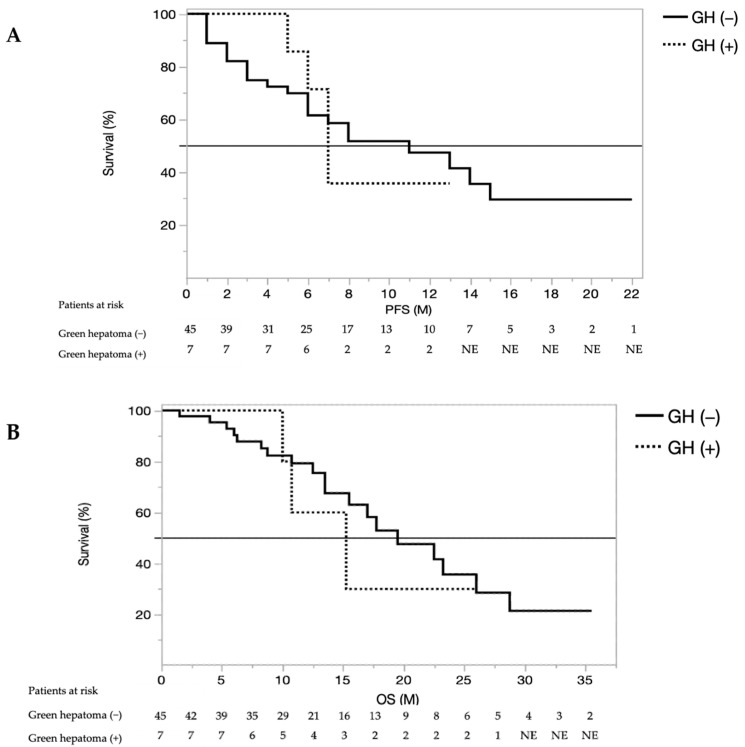
Kaplan-Meier estimates of (**A**) overall survival (OS) and (**B**) progression-free survival (PFS) in patients with unresectable HCC with or without iso-high intensity in the hepatobiliary phase of EOB-MRI. M, month; NE, not evaluated; GH, Green hepatoma.

Comparison of OS and PFS in patients with HCC with or without iso-high intensity in the hepatobiliary phase of EOB-MRI treated with lenvatinib are shown in Figure 3. PFS rates were not significantly different between patients with or without HCC with iso-high intensity in the hepatobiliary phase of EOB-MRI (median PFS; 7.0 and 8.3 months, respectively; HR = 1.041074, 95% CI: 0.863166–1.171921, *p* = 0.8456). Similarly, OS rates were not significantly different between patients with or without HCC with iso-high intensity in the hepatobiliary phase of EOB-MRI (median OS; 17.5 and 19.5 months, respectively; HR = 1.1449, 95% CI: 0.892235–1.090671, *p* = 0.8315). In addition, as shown in the Supplementary Table S1, cox regression analysis revealed that the presence of HCC with iso-high intensity in the hepatobiliary phase of EOB-MRI did not affect OS (HR 0.95, 95% CI 0.153–4.849, *p* = 0.9613).

### 3.4. Relationship between HCC with Iso-High Intensity in the Hepatobiliary Phase of EOB-MRI and WNT/β-Catenin Mutations Diagnosed by Cell-Free DNA

Finally, we analyzed the *CTNNB1* mutation profile in cfDNA in one patient with unresectable HCC with iso-high intensity in the hepatobiliary phase of EOB-MRI and three patients without HCC with iso-high intensity in the hepatobiliary phase of EOB-MRI who were treated with lenvatinib.

As shown in Table 4, of three the patient with HCC with iso-high intensity in the hepatobiliary phase of EOB-MRI, two (66.7% 2/3) showed *CTNNB-1* alterations (case 1; *CTNNB-1* T41A, variant frequency 30/13,721; *CTNNB-1* S45F, variant frequency 28/13,723, case 2; *CTNNB-1* T41A, variant frequency 196/12,329) in cfDNA.

In the four patients with HCC without iso-high intensity in the hepatobiliary phase of EOB-MRI, *CTNNB-1* genetic alterations were not detected in cfDNA.

## 4. Discussion

In this study, we revealed that the prevalence of HCC with high intensity in the hepatobiliary phase of EOB-MRI was 13.4% (7/52) among patients with unresectable HCC. All 7 patients had multiple HCCs and had both HCCs with or without iso-high intensity in the hepatobiliary phase of EOB-MRI simultaneously. Only 3 patients had multiple HCCs with iso-high intensity in the hepatobiliary phase of EOB-MRI. The response to lenvatinib, PFS, and OS was similar between patients with or without iso-high intensity in the hepatobiliary phase of EOB-MRI. Furthermore, our results supported the relationship between iso-high intensity in the hepatobiliary phase of EOB-MRI and *CTNNB-1* mutations detected in cfDNA.

The wnt/β-catenin pathway is known to be involved in the development of HCC. Hoshida et al. reported that HCC with WNT/β-catenin mutations is associated with good survival, pathologically well-differentiated tumors, smaller tumor sizes, and the S3 subclass [24]. Similarly, Zucman-Rossi et al. reported that HCC with WNT/β-catenin mutations is pathologically well-differentiated, lacks inflammatory infiltrates, and is classified as G5 and G6 subclass [25]. *CTNNB1* gene alterations in HCC are associated with less lymphocytic infiltration [25] and decreased levels of CCL5, which recruits antigen-specific CD8+ T cells, resulting in poor immunogenicity and resistance to ICI therapy [11,26]. Thus, HCC with *CTNNB1* alterations is called “cold tumors” or “immune desert tumors” [27]. Importantly, a recent report has revealed that HCC with mutant *CTNNB-1* has a poor response to ICI treatment, resulting in a poor prognosis [8].

However, because tumor biopsy is an invasive procedure in patients with unresectable HCC, it is difficult to obtain samples for genetic analyses. Thus, EOB-MRI is a potential alternative. In HCC, WNT/β-catenin mutations induce the upregulation of OATP1B3, which transports Gd-EOBDTPA [10], resulting in high intensity in the hepatobiliary phase of EOB-MRI [10]. Thus, HCC with iso-high intensity in the hepatobiliary phase of EOB-MRI indicates the presence of WNT/β-catenin mutations.

A previous study revealed that the prevalence of HCC with iso-high intensity in the hepatobiliary phase of EOB-MRI is 12% (22/180) to 20% (5/25) in typical resectable HCC [12,13,14,15]. To the best of our knowledge, this is the first report of the prevalence in patients with unresectable HCC who are adapted to systemic chemotherapy. Of 52 patients with unresectable HCC treated with lenvatinib, a total of 13% of patients had HCC with iso-high intensity in the hepatobiliary phase of EOB-MRI. This prevalence was similar to that reported in resectable HCC. However, all patients had multiple HCCs with and without iso-high intensity in the hepatobiliary phase of EOB-MRI. Only three patients had multiple HCCs with iso-high intensity in the hepatobiliary phase of EOB-MRI. This result might reflect the nature of multicentric carcinogenesis in HCC. This should be considered when evaluating the results of this study.

Comparison between patients with or without HCCs with iso-high intensity in the hepatobiliary phase of EOB-MRI revealed that in patients with HCCs with iso-high intensity in the hepatobiliary phase of EOB-MRI, the prevalence of HCC etiology of HBV had a low tendency (0% vs. 26.9% *p* = 0.0702), while the prevalence of HCC etiology of non-B non-C had a high tendency (85.7% vs. 53.3%, *p* = 0.0864). This result might be consistent with the previous report that the nonproliferation class of HCC had a high prevalence of WNT/b-catenin activation and etiology of HCV and alcohol, not that of HBV [28].

In this study, the response to lenvatinib (as evaluated by the DCR and ORR) was similar for patients with iso-high intensity in the hepatobiliary phase of EOB-MRI and without. In addition, OS and PFS were similar between patients with or without HCC with iso-high intensity in the hepatobiliary phase of EOB-MRI. These results are consistent with those of a previous report showing that sorafenib is similarly effective for patients with or without advanced HCC harboring a WNT/β-catenin mutation and that PFS and median OS after ICI treatment are lower in HCC with mutant WNT/β-catenin than with wild-type WNT/β-catenin [8]. Thus, in patients with unresectable HCC, EOB-MRI might be useful for selecting appropriate therapies. However, further studies are required, especially studies of the responses of unresectable HCC with iso-high intensity in the hepatobiliary phase of EOB-MRI to atezolizumab plus bevacizumab.

Finally, our analysis supported the relationship between unresectable HCC with high intensity in the hepatobiliary phase of EOB-MRI and *CTNNB-1* mutations determined by cfDNA analysis. The analysis of cfDNA is an effective method for the detection of genetic alterations in HCC [16,29,30], and it is less impacted by intratumoral heterogeneity than are analyses of single tumor tissue samples [31]. In three patients with unresectable HCC with high intensity in the hepatobiliary phase of EOB-MRI, *CTNNB1* alterations in cfDNA were observed in two patients (66.7%, 2/3), while all four patients without HCC with iso-high intensity in the hepatobiliary phase of EOB-MRI lacked mutations. Thus, *CTNNB1* alterations may be associated with HCC with iso-high intensity in the hepatobiliary phase of EOB-MRI in patients with resectable and unresectable HCC. In one patient with HCC with high intensity in the hepatobiliary phase of EOB-MRI, the CTNNB1 alteration in cfDNA was not detected. The reason why CTNNB1 alteration in cfDAA was not detected has not been clarified. Sensitivity of NGS analysis, quality of sample, or existence of another gene alteration of WNT/b-catenin pathway such as AXIN 1 may have been the reasons. Additionally, the number of patients was limited; thus, further studies of a larger number of patients are required.

Moreover, the prevalence of *CTNNB1* alterations in advanced HCC is reported to be 35.7% [8]. Thus, patients without HCC, iso-high intensity in the hepatobiliary phase of EOB-MRI might have *CTNNB1* alterations. Additional analyses of cfDNA for the detection of *CTNNB1* alteration are needed. However, in advanced HCC, multicentric carcinogenesis is usually observed, resulting in different genetic alterations within the same patient [32,33]. Thus, it is not clear whether genetic alterations in cfDNA represent all HCCs, and the results of genetic analyses should be interpreted with caution.

There are several limitations in this study. It was a retrospective single-center study and included a relatively small sample size, especially for patients who had HCC with iso-high intensity in the hepatobiliary phase of EOB-MRI. Moreover, the number of patients with an analysis for the genetic alteration of *CTNNB-1* in cfDNA was quite limited. Thus, those limitations should be considered when interpreting the results. However, this study is the first to estimate the prevalence of unresectable HCC with iso-high intensity in the hepatobiliary phase of EOB-MRI and its association with *CTNNB1* mutations and the response to lenvatinib. Therefore, future prospective multicenter studies, including large sample sizes, should be required to validate the results of this study. 

## 5. Conclusions

We revealed that the prevalence of HCC with iso-high intensity in the hepatobiliary phase of EOB-MRI in patients with unresectable HCC is 13.4% (7/52). Furthermore, we found that unresectable HCC with iso-high intensity in the hepatobiliary phase of EOB-MRI is related to *CTNNB-1* mutations in cfDNA, consistent with previous results for resectable HCC. The response to lenvatinib, PFS, and OS were similar in patients with or without iso-high intensity in the hepatobiliary phase of EOB-MRI. 

## Figures and Tables

**Table 1:** Characteristics of patients with unresectable HCC with or without iso-high intensity in the hepatobiliary phase of EOB-MRI.

Clinical Characteristics	Patients with HCC Treated by Lenvatinib (*n* = 52)	Green Hepatoma (*n* = 7)	Green Hepatoma (−) (*n* = 45)	*p*-Value
Baseline characteristics				
Age (yr)	70 (45–83)	70 (55–79)	71 (45–83)	0.9357
Sex (Male/Female)	46/6	7/0	39/6	0.3043
Etiology, No. (%)				
HBV	14 (26.9%)	0 (0%)	14 (31.1%)	0.0702
HCV	8 (15.3%)	1 (14.3%)	7 (15.5%)	0.9310
NBNC	30 (57.6%)	6 (85.7%)	24 (53.3%)	0.0864
Vascular invasion, No. (%)	16 (30.7%)	0 (0.0%)	16 (35.5%)	0.1006
Extrahepatic extension, No. (%)	16 (30.7%)	0 (0.0%)	16 (35.5%)	0.0580
BCLC stage, No. (%)				
B	27 (51.9%)	5 (71.4%)	22 (48.8%)	0.2669
C	25 (48.0%)	2 (28.6.%)	23 (51.1%)	
Child–Pugh class, No. (%)				
A	41 (78.8%)	4 (57.1%)	37 (82.2%)	0.1307
B	11 (21.1%)	3 (42.8%)	8 (17.7%)	
Biochemical analysis				
Albumin, g/dL	3.7 (2.8–4.6)	3.2 (2.9–4.6)	3.8 (2.8–4.6)	0.4354
Total bilirubin, mg/dL	0.7 (0.3–3.1)	1.2 (0.9–3.1)	0.7 (0.3–1.9)	0.0197
Prothrombin time, %	88.3 (40.7–117.7)	83.9 (46.6–107.6)	88.4 (40.7–117.7)	0.1637
Platelet, ×10^4^/μL	16.1 (4.4–51.7)	13.4 (4.4–51.7)	17.3 (6.5–51.7)	0.4448
Alpha-fetoprotein, ng/mL	78.3 (2.0–449,909)	2.2 (2.2–852.5)	78.3 (2.0–449,909)	0.4759
AFP, L3%	19.2 (0–99.5)	41.1 (0.5–80.4)	19.2 (0–99.5)	0.4273
PIVKA-II, mAU/mL	345 (13–93,644)	266 (26–29,756)	381 (13–93,644)	0.3961
RER	0.785 (0.446–1.446)	1.110 (1.018–1.446)	0.7526 (0.446–0.969)	<0.001

yr, years; HBV, hepatitis B virus; HCV, hepatitis C virus; NBNC, non-B non-C; BCLC, Barcelona Clinic Liver Cancer; AFP, alpha-fetoprotein; PIVKA-II, protein induced by vitamin K absence or antagonist-II; RER, relative enhancement ratio of nodule; Green hepatoma, HCC with iso-high intensity in the hepatobiliary phase of EOB-MRI.

**Table 2:** Characteristics of patients with HCC with iso-high intensity in the hepatobiliary phase of EOB-MRI.

Pt No.	Age (years)	Sex	HCCs in Liver (*n*)	Green Hepatoma (*n*)	Size of Green Hepatoma (mm)	RER (Median)	Etiology
1	55	M	6	3	12.6, 33.3, 32.5	1.155	HCV
2	79	M	5	2	11.1, 19.1	1.174	NBNC
3	70	M	3	2	16.6, 39.1	1.191	NBNC
4	73	M	3	1	10	1.073	NBNC
5	67	M	4	1	18.4	1.085	NBNC
6	83	M	5	1	19.8	1.105	NBNC
7	57	M	4	1	23.6	1.004	NBNC

RER, relative enhancement ratio of nodule; yr, years; M, Male; HCV, hepatitis C virus; NBNC, non-B non-C.

**Table 3:** Comparison of the response to lenvatinib between patients with or without unresectable HCC with or without iso-high intensity in the hepatobiliary phase of EOB-MRI imaging.

Treatment Response	Green Hepatoma	Green Hepatoma (−)	*p*-Value
	*n* = 7	*n* = 45	
CR/PR/SD/PD	0/3/4/0	4/19/16/6	0.5087
ORR	3 (42.8%)	22 (48.8%)	0.5901
DCR	7 (100%)	39(86.6%)	0.3416

CR, complete response; PR, partial response; SD, stable disease; PD, progressive disease; ORR, objective response rate; DCR, disease control rate.

**Table 4:** *CTNNB-1* gene alterations in cell-free DNA in five patients with or without iso-high intensity in the hepatobiliary phase of EOB-MRI.

Case	Green Hepatoma	Age (Years)	Sex	Etiology	Child–Pugh	BCLC	*CTNNB-1*	Mutation
1	+	70	M	NBNC	6	B	positive	T41AS45F
2	+	72	M	NBNC	6	B	positive	T41A
3	+	83	M	NBNC	6	B	negative	
4	−	70	M	NBNC	5	B	negative	
5	−	62	M	NBNC	6	B	negative	
6	−	70	M	NBNC	7	B	negative	
7	−	70	F	NBNC	5	B	negative	

BCLC, Barcelona clinic liver cancer; NBNC, non-B-non-C.

## Data Availability

The data that support the findings of this study are available from the corresponding author upon reasonable request.

## Supplementary Materials

The following are available online at https://www.mdpi.com/article/10.3390/cancers13143633/s1: Table S1; Cox regression analysis of association between clinical factors and overall survival.
