# Supplementary material for: Characteristics and Lenvatinib Treatment Response of Unresectable Hepatocellular Carcinoma with Iso-High Intensity in the Hepatobiliary Phase of EOB-MRI
Source: Cancers (Basel). 2021 Jul 20;13(14):3633. doi: 10.3390/cancers13143633 (PMC8304228; doi:10.3390/cancers13143633)
Supplement: Supplementary file 1 [file cancers-13-03633-s001.zip › cancers-1308213-supplementary.pdf]

cancers

MDPI

Supplementary Material

Characteristics and Lenvatinib Treatment Response of Unresectable Hepatocellular Carcinoma with Iso-High Intensity in the Hepatobiliary Phase of EOB-MRI

Akinori Kubo, Goki Suda, Megumi Kimura, Osamu Maehara, Yoshimasa Tokuchi, Takashi Kitagataya, Masatsugu Ohara, Ren Yamada, Taku Shigesawa, Kazuharu Suzuki, Naoki Kawagishi, Masato Nakai, Takuya Sho, Mitsuteru Natsuizaka, Kenichi Morikawa, Koji Ogawa, Shunsuke Ohnishi and Naoya Sakamoto

Table S1. Cox regression analysis of association between clinical factors and overall survival

Variables	Hazard Ratios	95% Confidence Interval	*p*-Value
Green hepatoma (+)	0.95	0.15-4.84	0.9613
Sex: Male	0.40	0.10-2.07	0.2527
HBV (+)	0.54	0.18-1.51	0.2459
HCV (+)	0.43	0.10-1.51	0.2013
NBNC (+)	1.27	0.43-4.68	0.5816
Child-Pugh B	1.48	0.81-8.51	0.5100
ALBI grade: 2	2.34	1.12-7.65	0.1173
BCLC stage: C	2.74	0.81-8.51	0.0251
Extrahepatic extension (+)	0.93	0.33-2.52	0.8882

HBV, hepatisis B virus; HCV, Hepatitis C virus; NBNC, Non-B and Non-C hepatitis; BCLC, Barcelona clinic liver cancer.
